# Data on children reentering foster care from kinship guardianship

**DOI:** 10.1016/j.dib.2018.04.022

**Published:** 2018-04-12

**Authors:** Arno Parolini, Aron Shlonsky, Joseph Magruder, Andrea Lane Eastman, Fred Wulczyn, Daniel Webster

**Affiliations:** a Department of Social Work, University of Melbourne, 161 Barry Street, Carlton, VIC 3053, Australia; b California Child Welfare Indicators Project, School of Social Welfare, University of California at Berkeley, 120 Haviland Hall, Berkeley, CA 94720-7400, USA; c Children's Data Network, School of Social Work, University of Southern California, 669W 34th Street, Los Angeles, CA 90089, USA; d Chapin Hall Center for Children, University of Chicago, 1313 East 60th Street, Chicago, IL 60637, USA

## Abstract

This article describes a dataset containing information on children exiting to kinship guardianship in California between 2003 and 2010 (*N* = 18,831). Children and young people in the sample were followed for up to fourteen years. The data presented here show summary statistics of the sample included in the analysis. Furthermore, the data consist of life tables showing counts of children at risk of reentry, counts of children who reentered the foster care system as well as nonparametric estimates of the survival function and the cumulative hazard function for the period 2003–2017.

**Specifications Table**

**Table:** 

Subject area	*Social Work*
More specific subject area	*Child Welfare*
Type of data	*Table, text file, figure, life table*
How data was acquired	*Data were extracted from the California Child Welfare Services/Case Management System, an administrative database maintained by the California Department of Social Services.*
Data format	*aggregated, processed, analyzed*
Experimental factors	*Sample construction involved quality assurance of data entries which resulted in the exclusion of three children due to missing or false information. Raw data were then aggregated to generate life tables, summary statistics and estimates of survival functions and hazard rates.*
Experimental features	*Statistical analysis including nonparametric survival analyses*
Data source location	*California, USA*
Data accessibility	*All data is with this article*
Related research article	*Parolini et al.*[1].

**Value of the data**

• Population-based dataset on children exiting to kinship guardianship in California between 2003 and 2010.
• Extensive follow-up period of up to fourteen years.
• The data analysis provides nonparametric estimates of the hazard of reentry into the foster care system.
• The data can be compared to data for other types of permanency.
• The data can be compared with data from other states or jurisdictions.

## 1 Data

The data presented in this article contain summary statistics and estimates of the survival function and the cumulative hazard function for children exiting to kinship guardianship between 2003 and 2010. The children in the sample were followed for up to fourteen years until they either reentered the foster care system or observations were censored. Censoring occurred on children's eighteenth birthday or January 1st, 2017, when the data were extracted. Overall, the dataset spans the period from 2003 to 2017. The data are presented in life tables for the total sample based on monthly intervals. Summary statistics for the underlying cohort are presented in Table 1. Additional details on the individual-level data and further analyses are presented in a companion article [1] and the corresponding supplementary material.

**Table 1:** Child characteristics of children exiting to kinship guardianship in California, 2003-2010.

Categorical Variables		No. of children^a^	%
Gender	Female	9,569	50.82%
Ethnicity	White	3,961	21.03%
	Black	5,884	31.25%
	Latino	8,237	43.74%
	Asian/Pacific Islander	437	2.32%
	Native American	312	1.66%
Disability diagnosed prior exit to kinship guardianship	Mental health	2,246	11.93%
	Other disability only	6,794	36.08%
Reason for removal	Neglect	15,533	82.49%
	Physical abuse	1,506	8.00%
	Sexual abuse	463	2.46%
	Other maltreatment	1,329	7.06%
Guardian	Single caretaker	13,452	71.44%

Continuous & Count Variables		No. of children	Mean (sd)

Age at exit to kin guardianship (years)		18,831	9.23 (4.588)
Number of previous episodes incl. base episode		18,831	1.31 (0.605)
Duration of base episode (months)^b^		18,831	38.70 (36.665)

Source: California Child Welfare Services/Case Management System, N=18,831

a Number of children for which the indicator variable takes the value of one.

b Base episode refers to the episode from which the child exited to kin guardianship.

## 2 Experimental design, materials, and methods

The data files were extracted from the California Child Welfare Services/Case Management System, a database containing information on the California child protection system, which is maintained by the California Department of Social Services. Data are available through a longstanding agreement between the University of California at Berkeley and the California Department of Social Services, which has ethical approval from the Institutional Review Board. After extraction, raw files underwent a quality assurance process and variables were recoded for analyses. In the following paragraphs we provide an overview of the variables included in the data described in this article.

*Months*: The variable of interest is the duration of kinship guardianship placement, the number of months that children spent in kinship guardianship. This variable was calculated as the difference between the date a child exited to kinship guardianship and the date the child either reentered the foster care system, turned eighteen, or was censored on January 1st 2017. While a child's reentry to foster care represents the event of interest, a child's 18th birthday is interpreted as a censoring date. The calculated difference in days was then transformed into monthly intervals [1].

*At risk*: This variable contains the number of children at risk of reentry at the beginning of each monthly interval. This is calculated as the number of children in kinship guardianship who have not reentered the foster care system in previous periods minus the number of children who were censored during the previous months, i.e., $n_{j}=n_{j-1}-{(d}_{j-1}+c_{j-1}),\;j=1,2,3,\ldots ,t$.

In the expression above, $n_{j}$ denotes the number of children at risk at the start of period *j*, *d*_*j*_ denotes the children who reentered to foster care during period *j*, and *c*_*j*_ denotes the censored observations during period *j*. Also note that $n_{0}=N$, the number of children at risk in the first period equals the total sample size.

*Reentries*: This variable contains the number of children who reentered to foster care during a particular period *j*. This count of guardianship discontinuities does not include positive reentries which either (i) maintained the guardianship with the same caregiver, (ii) resulted in adoption by the same caregiver or (iii) were caused by efforts towards reunification with a child's birth parents [1]. It is also important to note that only children's first reentry to foster care was considered, i.e., for children who had more than one episode of kinship guardianship only the first one was included in the data [1].

*Censored*: This variable contains counts of observations lost due to censoring during a particular period *j.* Censoring occurred on children's eighteenth birthday or if a child exited kinship guardianship for other reasons, e.g., on the date of finalization of adoption. In any case, all observations were censored latest on January 1st 2017, the date of data extraction.

*Survival*: The survivor function is estimated using the nonparametric estimator proposed by Kaplan and Meier [2], which does not make adjustments for interval censoring. This assumption has been assessed in the appendix to the companion paper of this article [1].

*Cum. Haz*: This variable contains estimates of the cumulative hazard function based on the Nelson-Aalen estimator [3].

The estimations of survivor and cumulative hazard functions were conducted using the *sts list* command in Stata SE 14.2 (StataCorp LP, College Station, TX). In addition to the variables listed above, standard errors and confidence intervals for the Kaplan-Meier and Nelson-Aalen estimators are also provided in the data [4].

As mentioned above, time is measured in months since exit to kinship guardianship. Overall, children exiting to kin guardianship between 2003 and 2010 had a restricted mean duration in kinship guardianship of 140.54 months (SE = 0.45), where the restricted mean duration was defined as ${\hat{\mu }}_{\mathrm{res}}=\int_{0}^{t_{\max}}S(t)\mathrm{dt}$ and $t_{\max}$ denoted the maximum observed time of reentry to foster care [4].

**Fig. 1:**
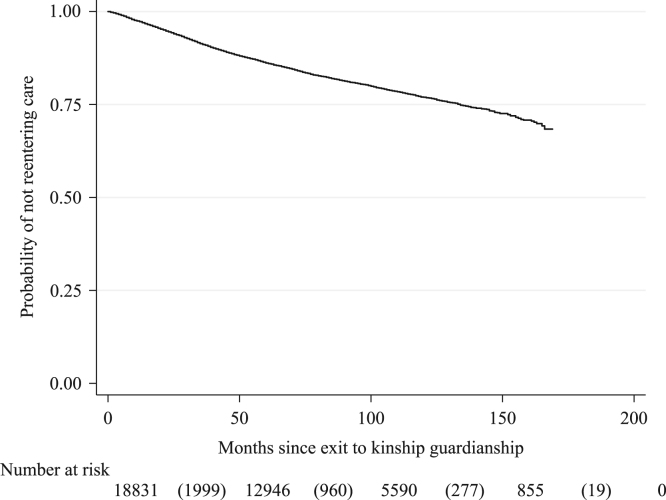
Kaplan-Meier survival function: children placed in kinship guardianship, 2003–2017. Cohort includes children who exited to kinship guardianship in California, 2003–2010. Source: California Child Welfare Services/Case Management System.

The Kaplan-Meier survival curve is plotted in Fig. 1 and shows that, in general, reentries to the foster care system were relatively rare. Of the total number of children exiting to kinship guardianship (*N* = 18,831), approximately 17.3% (*n* = 3255) of children reentered to foster care. As is shown in the risk table at the bottom of Fig. 1, 61.41% (*n* = 1999) of all observed negative reentries in the dataset occurred within 50 months (4.2 years), whereas 9.09% of all observed reentries occurred after 100 months (approx. 8 years).

**Fig. 2:**
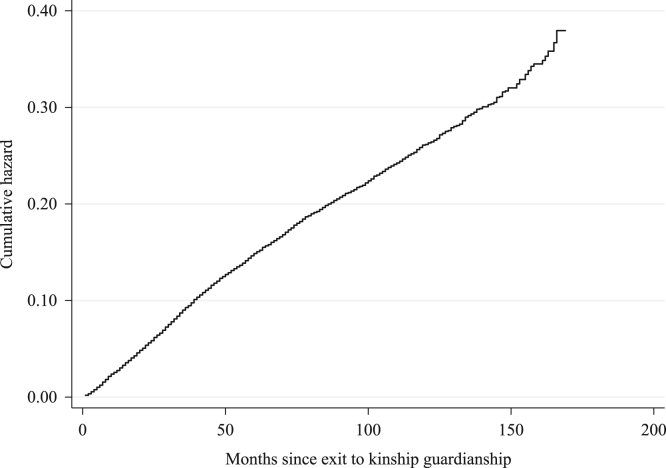
Nelson-Aalen cumulative hazard function: children placed in kinship guardianship, 2003–2017. Cohort includes children who exited to kinship guardianship in California, 2003–2010. Source: California Child Welfare Services/Case Management System.

To investigate whether the hazard rate changed over time, we also plot the cumulative hazard function using the Nelson-Aalen estimator (Fig. 2). There appeared to be a decrease in slope around 50 months, a slight increase at approximately 100 months and a steep increase just after 150 months. These changes in the slope of the cumulative hazard function indicate an increasing hazard rate in the right tail of the function. Based on the cumulative hazard function in the Supplementary tables, one can also estimate the hazard function using a kernel smoother [4], which is illustrated in the companion paper [1]. Based on these results, it becomes obvious that the hazard function of reentry into foster care is bi-modal, having maxima around 35 months (about 3 years) and at approximately 145 months (12 years).
