# Supplementary material for: Data on children reentering foster care from kinship guardianship
Source: Data Brief. 2018 Apr 12;18:1457–61. doi: 10.1016/j.dib.2018.04.022 (PMC5997012; doi:10.1016/j.dib.2018.04.022)
Supplement: Supplementary file 1 — Transparency document [file mmc1.docx]

**Data on children reentering foster care from kinship guardianship**

**Declaration of interest:**

The authors confirm that they have no conflicts of interests to declare.

All authors have read and approved the final version of this article.

This research did not receive any specific grant from funding agencies in the public, commercial, or not-for-profit sectors.

We confirm that this research was conducted with ethical approval of all relevant bodies and that such approvals were acknowledged within the manuscript
